# Mapping risk of gambling disorder in older adults: a neural network approach

**DOI:** 10.3389/fpsyt.2026.1936618

**Published:** 2026-10-05

**Authors:** Roser Granero, Fernando Fernández-Aranda, Isabel Baenas, Lucero Munguía, Anahí Gaspar-Pérez, Mónica Gómez-Peña, Jeffrey Derevensky, Sally Gainsbury, Rebeca Rodríguez-García, Susana Jiménez-Murcia

**Affiliations:** 1 Department of Psychobiology and Methodology, Universitat Autònoma de Barcelona - UAB, Barcelona, Spain; 2 Ciber Fisiopatología Obesidad y Nutrición (CIBERobn), Instituto Salud Carlos III, Madrid, Spain; 3 Group of Psychoneurobiology of eating and addictive behaviors, Institut d’Investigació Biomèdica de Bellvitge - IDIBELL, L’Hospitalet de Llobregat, Spain; 4 Department of Clinical Psychology, Hospital Universitari de Bellvitge, L’Hospitalet de Llobregat, Spain; 5 Department of Clinical Sciences, School of Medicine and Health Sciences, Universitat de Barcelona - UB, L’Hospitalet de Llobregat, Spain; 6 International Centre for Youth Gambling Problems and High-Risk Behaviors, McGill University, Montreal, QC, Canada; 7 School of Psychology, Brain & Mind Centre, Science Faculty, University of Sydney, Sydney, NSW, Australia

**Keywords:** gambling disorder, multilayer perceptron, neural network, older adults, predictive models

## Abstract

**Background and objectives:**

Gambling disorder (GD) in older adults remains insufficiently understood, despite growing concerns about gambling-related harm in older adulthood. Risk profiles and clinical presentations in this age group may differ from those typically described in younger or mixed-age samples, reflecting age-specific psychosocial contexts, health conditions, and life transitions. Traditional statistical approaches may be limited in their ability to model the complex, non-linear interplay among psychological, behavioral, and contextual factors. The present study aimed to apply neural network–based models to predict the presence and severity of GD in older adults.

**Methods:**

The study used a cross-sectional, single-center, case-control design. Data were obtained from adults aged 50 to 85 years, with 50 years adopted as a pragmatic lower threshold given the lack of consensus on defining older adulthood, comprising a clinical sample of 745 patients seeking treatment for gambling-related problems at a specialized treatment hospital unit, and a healthy control (HC) group of 136 participants without gambling-related problems, recruited at non-psychiatric hospital outpatient services.

**Results:**

A neural network (multilayer perceptron, MLP) with one hidden layer composed of eight neurons showed excellent discrimination between individuals with GD and healthy controls in the complete sample (AUC = 0.987, 90.4% specificity, 97.7% sensitivity, and 96.6% classification accuracy). In the independent holdout subset, the model yielded an AUC of 0.890, with 75.0% specificity, 96.2% sensitivity, and 93.3% classification accuracy (although these estimates should be interpreted cautiously because the holdout included only 12 healthy controls). Novelty seeking emerged as the strongest contributor to the model, followed by emotion regulation difficulties, particularly non-acceptance of emotional responses and reduced impulse control. Focusing exclusively on the clinical GD subsample, a second MLP including six hidden neurons indicated that overall psychological distress was the primary factor associated with disorder severity. Additional relevant contributors to GD included novelty seeking, gambling-related cognitive distortions, and lower self-directedness.

**Conclusion:**

The neural network models effectively classified GD and its severity in older adults, identifying a multifactorial pattern characterized by affective distress, emotion regulation difficulties, and distorted gambling-related beliefs. These findings highlight the potential of non-linear modeling to characterize patterns associated with GD in older adults, while the clinical utility of machine learning tools for age-sensitive detection and intervention remains preliminary and requires further validation.

## Highlights

- Neural networks accurately classified older adults with gambling disorder versus healthy controls.
- Multivariate patterns characterized gambling disorder in older adults.
- Psychological distress was the strongest predictor of gambling disorder severity among treatment-seeking older adults.
- Neural networks showed advantages over conventional logistic and ordinal regression models.
- Neural networks identified multivariate contributions across clinical and psychopathological variables.

## 1 Introduction

Gambling disorder (GD) is a behavioral addiction characterized by persistent and recurrent maladaptive patterns of gambling behavior that lead to clinically significant distress or impairment. In the *Diagnostic and Statistical Manual of Mental Disorders, Fifth Edition* (DSM-5; 1), GD is classified within the section “Substance-Related and Addictive Disorders,” reflecting its conceptualization as a behavioral addiction sharing core features with substance use disorders. According to the DSM-5, GD involves loss of control over gambling, increasing preoccupation with betting activities, chasing losses, and continuation of gambling despite negative personal, social, or financial consequences. Similarly, the *International Classification of Diseases, Eleventh Revision* (ICD-11; 2) includes GD under “Disorders due to addictive behaviors,” emphasizing impaired control over gambling, increasing priority given to gambling over other activities, and persistence or escalation of gambling despite adverse consequences. Although traditionally studied in younger and middle-aged populations, GD is increasingly recognized as a significant mental health concern in older adults, where it may interact with age-related psychological, social, and health-related changes in ways that remain insufficiently understood.

### 1.1 Gambling disorder in older adults: risk factors and age-related considerations

Epidemiological evidence indicates that participation in gambling activities among older adults can reach as high as 85.6% in some populations and cultural contexts (3). The prevalence of lifetime gambling disorder among adults aged 60 years and older varies widely across studies, with estimates ranging from 0.01% to 10.6% (4). This variability in the prevalence estimates can be attributed to multiple factors, including differences in sample composition (e.g., age groups, population- versus clinical-based samples, and sociodemographic characteristics), the measurement instruments used (screening tools versus diagnostic assessments), the availability of different forms of gambling, and the definition of the construct itself (GD versus problem gambling). Within older adults, some studies have reported higher prevalence rates among the younger segment of older adults than among the oldest age groups. Global meta-analytic findings further suggest that GD is less prevalent among older adults than among younger cohorts. For example, older adults have been reported to be less likely to report problem gambling than middle-aged adults (odds ratio [OR] = 0.80; 5). Additionally, men consistently show higher rates of gambling-related problems than women (4, 6). A recent study of older adults similarly reported higher prevalence among men than women (9.6% vs. 0.9%; 7). Nevertheless, rates of gambling-related problems among women (particularly in older adults) may be higher than reported, as factors such as stigma, shame, and barriers to seeking treatment can lead to an underestimation and a distorted understanding of gambling disorder (GD) in women (8–10).

Regarding vulnerability factors, older adulthood is characterized by a range of transitions that may be associated with greater vulnerability to problem gambling (10). Retirement, bereavement, social isolation, declining physical health, fixed or reduced income, loss of daily structure, and the loss of a spouse or partner (particularly when followed by complicated grief) can contribute to emotional distress and a search for stimulation, distraction, or social contact, which in turn may influence gambling involvement in some older adults, potentially as a coping strategy (e.g., older adults may gamble more to ameliorate negative affective states or to fill gaps in previously enjoyed activities) (11–16).

In addition to these psychosocial transitions, normative neurocognitive changes that accompany aging may further be relevant to gambling-related decision-making and behavior (17). In terms of cognitive functioning, aging is associated with changes in several interconnected cognitive domains (18): a) processing speed and working memory, which can affect the efficiency with which information is processed and integrated during decision-making; b) decreases in inhibitory control, that may allow distracting or irrelevant information to influence choices, leading to greater reliance on habitual or automatic responses; and c) reductions in executive functioning, that can impair the efficient allocation of cognitive resources across the different stages of complex tasks. Together, declines in processing speed, working memory, and executive control can affect the ability to integrate multiple sources of information, evaluate risks and consequences, and make adaptive decisions (19, 20). These cognitive changes are particularly relevant to GD, as effective gambling-related decision-making requires the ability to recognize and respond to losses, inhibit impulsive bets, and adjust gambling behavior in the face of negative outcomes. Alterations in reward processing (21) may further increase the appeal of gambling as a source of stimulation or immediate gratification, while reduced cognitive control can allow distractions or irrelevant cues to bias decision-making. Overall, these age-related cognitive changes may be relevant to the initiation, persistence, or severity of problematic gambling behavior in some older adults.

Related to age-related cognitive vulnerabilities, older adults may be particularly susceptible to gambling-related cognitions, described as distorted beliefs about gambling outcomes that reinforce risky gambling behavior. Cognitive distortions such as *illusion of control*, *interpretive bias*, *superstitious beliefs*, and *chasing wins or losses* have been identified as gambling-related cognitions and behaviors associated with greater gambling problems in older adults despite negative consequences (22). Empirical data also indicate that age interacts with cognitive biases and impulsivity in GD, such that both younger and older gamblers exhibit high levels of irrational beliefs and decision-making biases, which are directly associated with greater gambling severity (23, 24). Furthermore, preliminary experimental evidence suggests that attentional biases and craving linked to gambling cues are prominent in older gamblers, with older adults showing difficulty disengaging attention from gambling-related stimuli, a factor that may further reinforce distorted cognitions and problem behavior (25). These findings underscore the importance of addressing *gambling-related cognitive distortions* in older populations, as such cognitions may be relevant to understanding the persistence and severity of gambling problems, particularly when considered alongside age-related changes in cognitive functioning.

GD in older adults has been associated with substantial comorbidity with physical and mental health problems, and this comorbid pattern has been associated with a lower quality of life in medical, social, and emotional domains when compared with social gamblers or non-gamblers (16, 17). Broader epidemiological evidence has revealed that problem gambling and GD in older adults are associated with increased psychiatric comorbidity, including substance use, mood, and anxiety disorders (3, 26). Prospective evidence indicates that the severity of comorbid disorders may be associated with changes in gambling activity, with greater severity of depression and post-traumatic stress disorder associated with increased gambling, whereas greater severity of agoraphobia, social phobia, substance use disorders, and compulsive computer use has been associated with reduced gambling activity (27). In addition, research on older adults treated with dopaminergic medications (especially dopamine agonists used in Parkinson’s disease) has documented the emergence of impulse control disorders as an adverse effect of pharmacotherapy, indicating an association between medication-related impulsivity and gambling problems in susceptible individuals (28, 29).

At the same time, older adults are not a homogeneous group, and individual differences in personality and emotional functioning may play an important role in individual differences in GD presentation and severity (30). Traits such as impulsivity, sensation seeking, and low self-directedness have been linked to addictive behaviors across the lifespan, and research specifically with older gamblers indicates that impulsivity and related cognitive control processes are elevated in GD and are associated with neural markers of impulsive behavior (e.g., higher impulsivity scores and altered prefrontal serotonergic function in older GD patients compared with age-matched controls) (31). Similarly, difficulties in emotion regulation may be particularly relevant in older adults facing cumulative stressors, personal loss, and health concerns. Emotion regulation has also been associated with GD, with clinical evidence indicating a direct association between emotion regulation difficulties and greater disorder severity (32). In the same study, participants in the older subgroup, defined according to the sample median age rather than an established older-adult threshold, showed higher levels of non-acceptance of emotions, limited access to emotion regulation strategies, lack of emotional clarity, and overall emotion regulation difficulties than younger participants; however, severity was higher among younger participants, and emotion regulation difficulties did not mediate the association between age and GD severity.

### 1.2 Predictive modeling of GD severity in older adults

Despite the concern and recognition of GD among older adults, research has predominantly focused on prevalence estimates and descriptive clinical features, with comparatively less attention paid to the complex interplay among sociodemographic factors, personality traits, emotional processes, and broader mental health issues (14). GD among older adults can have substantial social and economic consequences that extend beyond the individual, including effects on family members and other affected others (33). Consequently, gambling-related harms in older adults may contribute to increased public expenditures and place additional burdens on family support systems (34). Understanding variability in both the onset and severity of GD is particularly important, yet challenging, because the disorder is shaped by multiple interacting domains, including sociodemographic characteristics, psychiatric comorbidity and general psychological distress, maladaptive personality traits, emotion regulation deficits, and gambling-specific cognitive distortions. Identifying how these factors combine predicting disorder development and clinical presentation is critical for accurate stratification of at-risk individuals and for tailoring interventions in older adults, including concerned significant others (35). Moreover, GD may interact with age-related health problems and reduced social or financial resources, thereby exacerbating negative outcomes and complicating clinical management (3, 17).

Traditional statistical approaches have provided valuable insights into individual correlates of GD; however, their ability to capture complex, non-linear relationships and interactions among multiple psychological and socio-contextual variables may depend on the specific model and assumptions adopted (36, 37). Predictive modeling techniques based on machine learning, such as neural networks, may be able to capture clinically meaningful patterns that characterize subgroups of individuals with differing levels of risk and disorder severity. Machine learning models have been increasingly applied in clinical research to improve prediction of complex outcomes and to refine prediction models by integrating multidimensional data sources (38).

Neural networks may improve the identification of high-risk profiles/patterns, ultimately supporting more personalized and efficient clinical decision-making, because they can simultaneously process a large number of interrelated variables and detect non-linear relationships that may not be apparent using conventional methods (39). Structurally, these models consist of interconnected layers of computational units (“neurons”) that transform input data through weighted connections and non-linear activation functions. During training, the network iteratively adjusts these weights to minimize prediction error, enabling it to learn patterns that link combinations of sociodemographic, psychological, and clinical features with both the presence and severity of GD. By learning directly from the data rather than relying on pre-specified interaction terms, neural networks can uncover latent structures that reflect how multiple domains jointly contribute to disorder risk and clinical expression (40, 41). This integrative, data-driven approach may help characterize profiles/patterns associated with different levels of GD severity and generate hypotheses for future research on risk stratification and clinical assessment (42).

Although machine learning approaches have already been applied to gambling-related research, previous studies have primarily focused on identifying high-risk or problem gambling, predicting gambling-related behaviors, or examining gambling risk factors. For example, data-mining and supervised machine-learning approaches, including artificial neural networks, have been used to classify high-risk online gamblers and predict self-exclusion and gambling risk, while more recent work has applied machine-learning techniques to the identification of problem gamblers (43–46). A recent scoping review further highlights the growing application of data-science methods in gambling research (47). However, the application of neural network models to the characterization of GD presence and severity in treatment-seeking older adults remains limited, particularly when integrating sociodemographic, clinical, personality, emotion-regulation, psychopathological, and gambling-related cognitive variables.

### 1.3 Justification and objectives of the study

Against this background, there is a clear need for studies that simultaneously examine socio-contextual characteristics, personality traits, emotion regulation processes, general psychopathology, and gambling-related cognitions in older adults, using analytical approaches capable of modeling their joint and potentially non-linear effects.The present study addresses these gaps by applying neural network models to a comprehensive set of psychological and sociodemographic variables in a sample of adults aged 50 to 85 years. Two complementary aims were pursued. First, we sought to develop a model capable of accurately discriminating individuals with GD from healthy controls and to identify the psychological patterns underlying this classification. Second, we aimed to predict the severity of GD among affected individuals and to characterize the patterns associated with higher versus lower levels of clinical severity.

## 2 Methods

This study was reported in accordance with the TRIPOD+AI statement for studies developing and validating artificial intelligence-based prediction models (48).

### 2.1 Participants

This cross-sectional, single-center study used a case-control comparison between a clinical group of treatment-seeking patients with GD and a separately recruited healthy control (HC) group. The clinical GD group comprised 745 treatment-seeking patients aged 50 to 85 years who attended the Behavioral Addictions Unit of the Department of Clinical Psychology at the University Hospital of Bellvitge (Barcelona, Spain). This hospital is a tertiary referral center with specialized expertise in the assessment and treatment of behavioral addictions and receives referrals from across the metropolitan Barcelona area and even from the whole region. Recruitment took place over an extended period, from January 2014 to December 2025.

Inclusion was based on seeking treatment for gambling-related problems. Only data collected during the initial pre-treatment clinical assessment were used for the present analyses. These evaluations formed part of routine clinical practice and were conducted by experienced clinicians, who ensured the completeness of assessment protocols and clarified any uncertainties during administration. Participants were excluded if clinically relevant neuropsychological or psychiatric conditions were identified that could substantially interfere with the assessment or the validity of their responses, including major neurodegenerative disorders or acute psychotic states. Similarly, participants who were unable to adequately understand or complete the assessment because of language barriers were not included. The assessment was supervised by experienced clinicians, who also evaluated whether participants were able to understand the procedures, provide valid responses and complete all the questionnaires. All patients provided written informed consent permitting the use of their clinical data for research conducted within the unit. To ensure consistency across the recruitment period, the same diagnostic criteria and assessment instruments were applied throughout.

The HC group consisted of 136 individuals within the same age range, recruited from broader research projects investigating risk factors for problematic gambling in the general population. HC participants were recruited from the same geographical catchment area as the clinical sample and were individuals recruited at non-psychiatric hospital outpatient services at the same Hospital (Bellvitge University Hospital), concretely individuals attending the Dentistry and Podiatry services for reasons unrelated to gambling problems. These services do not primarily provide care for gambling-related disorders or severe psychiatric conditions. This recruitment strategy helped reduce potential differences related to geographical origin and recruitment setting. Data collection was supervised by senior researchers, who explained the study objectives and verified that questionnaires were completed in accordance with study procedures. Participation was voluntary, and no financial or academic incentives were provided. For HC, the same general requirement for adequate understanding and completion of the assessment was applied. Participants unable to understand the study procedures or complete the assessment adequately were not included.

The selection of 50 years as the lower age threshold for defining older adulthood in the present study reflects the absence of a universally accepted cutoff in the literature. Chronological age boundaries for older adults vary depending on the conceptual framework and the phenomena under investigation, encompassing sociodemographic considerations (e.g., retirement age and role transitions), neurocognitive changes (such as the age at which subtle cognitive declines may become more apparent at the population level), and the clinical characteristics of specific disorders. In the field of GD, inconsistent definitions of “older adults” across studies have hindered the comparability of results. Previous studies have used different age thresholds to define older adults, with lower age limits ranging from approximately 45 years (5) to 50 years (30, 31). In this context, we have selected the 50-year threshold because it represents a pragmatic and theoretically informed compromise that captures individuals likely to be experiencing age-related psychosocial and health changes while still allowing for sufficient variability to examine risk and severity patterns within older adults.

### 2.2 Measures

The assessment protocol differed between the GD and HC groups because HC participants were recruited through several research projects conducted over different periods, in which the specific measures administered were not identical. Given the longitudinal and multi-project nature of HC recruitment, we prioritized measures that were available for a sufficiently large proportion of controls and that allowed the largest possible HC sample with comparable demographic and geographical characteristics to be retained. Consequently, measures collected only in a limited subset of HC participants, including the UPPS-P and the Gambling-Related Cognitions Scale (GRCS), were not included in the HC assessment set and were therefore analyzed only within the GD group.

#### 2.2.1 Data collection procedures for the overall sample (GD and HC)

Sociodemographic variables. Information on participants’ background was collected, including age, sex, country of birth (Spain vs. other), and socioeconomic status (SES). SES was estimated using the Hollingshead Social Position Index (49), which combines educational attainment and occupational status to provide a standardized indicator of family socioeconomic standing suitable for cross-study comparisons.

Symptom Checklist-90-Revised (SCL-90-R). Psychological distress and psychopathological symptoms were assessed using the 90-item SCL-90-R (50), which covers nine domains: somatization, obsessive–compulsive symptoms, interpersonal sensitivity, depression, anxiety, hostility, phobic anxiety, paranoid ideation, and psychoticism. Three global indices are also derived: the Global Severity Index (GSI), reflecting overall symptom burden; the Positive Symptom Total (PST), representing the number of reported symptoms; and the Positive Symptom Distress Index (PSDI), capturing symptom intensity. The Spanish version of the instrument was used (51), showing good internal consistency in the current sample (from α = 0.778 for paranoid ideation to α = 0.980 for the global scale).

Difficulties in Emotion Regulation Scale (DERS). Emotion regulation was evaluated using the 36-item DERS (52), measuring six facets: limited awareness, lack of clarity, non-acceptance of emotions, difficulties in goal-directed behavior when distressed, limited access to regulation strategies, and impulse control problems. A total score indicates overall dysregulation. Validated Spanish versions were administered (53, 54), with internal consistency in this sample ranging from α = 0.721 (lack of clarity) to α = 0.926 (total scale).

Temperament and Character Inventory–Revised (TCI-R). Personality traits were assessed using the 240-item TCI-R (55), measuring four temperament dimensions (novelty seeking, harm avoidance, reward dependence, persistence) and three-character dimensions (self-directedness, cooperativeness, self-transcendence). The Spanish validated version (56) was administered, with internal consistency ranging from α = 0.756 (cooperativeness) to 0.867 (persistence).

#### 2.2.2 Measures specific to the GD group

*Diagnostic Questionnaire for Gambling Disorder.* GD symptoms were assessed using the Spanish adaptation (57) of the Diagnostic Questionnaire for Pathological Gambling originally developed by Stinchfield (58). The instrument consists of 19 dichotomous (yes/no) items derived from the DSM-IV pathological gambling criteria (59). While the questionnaire was administered in its original format, scoring was adapted to DSM-5 criteria (1), excluding the two items corresponding to the DSM-IV illegal acts criterion, which was removed in DSM-5. The resulting nine binary criterion indicators were summed to obtain a DSM-5 GD severity score. The questionnaire was administered to participants in both the GD and HC groups. In the GD group, the total number of endorsed DSM-5 criteria was used as the severity outcome in the clinical analyses. In the HC group, the absence of GD was established by verifying that participants endorsed none of the DSM-5 criteria for GD. The scale demonstrated acceptable reliability in this sample (Cronbach’s α = 0.745), calculated using the nine derived DSM-5 criterion indicators.

UPPS-P Impulsive Behavior Scale. Impulsivity traits were measured with the 59-item UPPS-P (60), assessing five dimensions: negative urgency, positive urgency, sensation seeking, lack of premeditation, and lack of perseverance. A total score can also be calculated. The Spanish adaptation was employed (61), with reliability estimates ranging from α = 0.767 (lack or perseverance) to α = 0.915 (total scale).

Gambling-Related Cognitions Scale (GRCS). Gambling-related cognitive distortions were measured with the 23-item GRCS (62), covering interpretative bias, illusion of control, predictive control, gambling expectancies, and perceived inability to stop gambling. The Spanish version (63) was used, showing adequate psychometric performance (from α = 0.763 for illusion of control to α = 0.926 for total scale).

Gambling-related clinical variables. Structured clinical interviews were conducted with GD participants to collect detailed information on gambling behavior, including age at onset, duration of problems, and substance use (tobacco, alcohol, other drugs).

### 2.3 Statistical analyses

Statistical analyses were conducted with IBM SPSS Statistics v29 (Windows). Neural network–based predictive modeling was implemented using the Multilayer Perceptron (MLP) procedure, a supervised learning technique widely applied in mental health research for pattern recognition and outcome classification. The full dataset was randomly partitioned into three subsamples. The partitioning was performed automatically by the MLP procedure and was conducted independently for each model. No diagnostic or clinical variable was specified for stratification. Sixty percent of cases were assigned to the training set, which was used to estimate network parameters by iteratively adjusting connection weights and biases to minimize prediction error. Thirty percent constituted the testing set, which served to evaluate model performance on unseen data during development and to monitor model performance during training (e.g., early stopping or hyperparameter tuning). The remaining 10% was assigned to the holdout set, which was reserved for a final evaluation of the trained network on previously unused data. The holdout set was not used for model development, architecture selection, or tuning and remained untouched until the final evaluation of the selected network. Neurons in the hidden layers used the hyperbolic tangent activation function, whereas the Softmax function was applied in the output layer. Network architecture (i.e., number of hidden layers and units) was selected automatically by the MLP procedure during model development, rather than being manually specified by the researchers. The number of hidden units was constrained between 1 and 50. Training was subject to predefined stopping criteria based on changes in prediction error and maximum training time. No additional hyperparameter optimization was performed beyond the automatic architecture selection implemented by the MLP procedure. Reproducibility was ensured by fixing the random number generator with the SPSS command SET SEED = 20261502 prior to model estimation. No missing data were present in the database used for analysis. All assessments were completed during scheduled in-person sessions conducted at the hospital under the supervision of experienced clinicians, who were available to clarify questionnaire items and review assessment protocols for completeness at the time of data collection. Consequently, no imputation procedures were required. Predictors were specified *a priori* based on their sociodemographic, clinical, psychopathological, behavioral, and personality relevance to the outcomes. All prespecified predictors were entered into the corresponding MLP model; no stepwise or other data-driven variable-selection procedure or separate feature-selection procedure was applied. Continuous variables were entered using their original measurement scales, without prior standardization or transformation. Binary categorical variables were coded as 0/1 (categorical variables with more than two categories were transformed into binary 0/1 indicators). No specific regularization method or formal multicollinearity-based variable exclusion procedure was applied.

The first neural network was trained using the complete sample to discriminate between diagnostic groups (output coding: 1 = GD, 0 = HC). Input variables included sociodemographic characteristics (age, marital status, education level, employment status, social position index, and country of origin), psychopathological symptom dimensions (SCL-90-R subscales), emotion regulation difficulties (DERS dimensions), and personality traits (TCI-R dimensions). Classification performance was examined separately in the training, testing, and holdout subsets, as well as for the complete analytical sample. Sensitivity (Se) represented the proportion of participants with GD correctly classified by the model, while specificity (Sp) indicated the proportion of healthy controls accurately identified as not having gambling-related problems. To provide a more comprehensive assessment of classification performance in the presence of class imbalance, positive predictive value (PPV), negative predictive value (NPV), F1 score, and balanced accuracy were also calculated. PPV represented the proportion of participants classified as GD who were correctly classified, whereas NPV represented the proportion of participants classified as HC who were correctly classified. The F1 score was calculated as the harmonic mean of PPV and sensitivity, and balanced accuracy was calculated as the arithmetic mean of sensitivity and specificity. Overall accuracy was also reported as the proportion of all participants correctly classified by the model. Discriminative capacity was further examined through Receiver Operating Characteristic (ROC) curve analysis and the corresponding Area Under the Curve (AUC). The ROC curve depicts the trade-off between sensitivity (true positive rate) and 1 − specificity (false positive rate) across all possible probability thresholds generated by the neural network, using observed clinical status (GD vs. HC) as the reference standard. ROC analysis was used to quantify the model’s ability to discriminate between the GD and HC groups in the present case-control sample. The AUC provides a global measure of discrimination between the two study groups across the range of predicted probabilities (64), ranging from 0.5 (chance-level discrimination) to 1.0 (perfect discrimination). Following previously published interpretative guidelines, AUC values < 0.70 were considered indicative of poor discrimination, values between 0.70 and <0.80 of fair discrimination, values between 0.80 and <0.90 of good discrimination, and values ≥ 0.90 of excellent discrimination (65). ROC analysis was initially conducted using the predicted probabilities for the complete analytical sample. To further assess model discrimination in previously unused data, an additional ROC analysis was performed restricted to the holdout subset. Cumulative gains charts were also inspected to assess practical utility, estimating the proportion of true GD cases captured as a function of the proportion of individuals selected based on predicted probabilities. Moreover, to evaluate the stability and robustness of the neural model beyond the original single random partition, an additional internal validation procedure based on repeated random splits was conducted. Specifically, the complete modeling process was repeated across 100 independent random partitions of the dataset, each using the same allocation scheme (60% training, 30% testing, and 10% holdout), predictor set, network architecture selection procedure, and training parameters. For each iteration, model performance was evaluated exclusively in the holdout subset. The distribution of AUC, Se, Sp, and overall accuracy across the 100 repetitions was summarized using the minimum, maximum, mean, standard deviation (SD) and coefficient of variation (CV, calculated as a relative indicator of performance stability across repeated data partitions).

A second neural network model was estimated exclusively in the GD subsample to predict disorder severity, operationalized as the number of DSM-5 criteria endorsed (output variable). Predictors comprised sociodemographic variables (age, marital status, education level, employment status, social position index, country of origin, and gender), together with gambling-related clinical and behavioral indicators (age of onset, duration of problematic gambling, participation in strategic games, and online gambling involvement), substance use (tobacco, alcohol, and other illicit drugs), global psychological distress (SCL-90-R Global Severity Index), overall impulsivity (UPPS-P total score), global emotion regulation difficulties (DERS total score), gambling-related cognitive distortions (GRCS total score), and personality traits (TCI-R dimensions). Model fit was examined using a predicted-versus-observed plot, allowing visual inspection of agreement between estimated and actual severity scores across the full outcome range and facilitating the detection of systematic overestimation or underestimation. A detrended normal Q–Q plot of standardized predicted values was analyzed to evaluate departures from normality and to identify potential distributional anomalies or influential outliers. Additionally, model performance was evaluated using the adjusted coefficient of determination (adjusted R²), root mean square error (RMSE) and mean absolute error (MAE). To further assess the robustness and stability of the model, an internal validation procedure based on 100 repeated random splits was conducted using the same resampling strategy described for the first neural network model. For each iteration, model performance was evaluated in the holdout subset using adjusted R², RMSE, and MAE, and the distribution of these indices across repetitions was summarized to assess the stability and generalizability of the severity prediction model.

The study used a convenience sample comprising all available participants who met the eligibility criteria. The GD-versus-HC classification model included 28 input variables for 881 participants (745 GD and 136 HC), whereas the GD severity model included 25 predictors among the 745 participants with GD. The relationship between sample size and number of predictors was approximately 31.5 participants per input variable for the classification model and 29.8 participants per predictor for the severity model. These ratios indicate a favorable relationship between the available sample size and the dimensionality of the input data. However, simple participant-to-predictor ratios do not provide a formal criterion for determining sample-size adequacy in prediction models, as sample-size requirements depend on factors such as the number of predictor parameters, outcome proportion, and expected model performance (66). Therefore, these ratios are reported descriptively rather than as a formal sample-size justification. To provide a more rigorous assessment, sample size adequacy for the GD-versus-HC classification model was additionally evaluated according to the framework proposed by Riley et al. (67) for prediction models with binary outcomes. Using the observed outcome prevalence (15.4%), 28 candidate predictor parameters, and the estimated Cox-Snell R² of the holdout set (0.39), the minimum required sample size was estimated at 750 participants, including 116 outcome events. The available sample of 881 participants therefore exceeded this requirement, supporting the adequacy of the sample size for developing the prediction model.

The predictors considered in the study were selected based on their theoretical and empirical relevance to GD, including demographic, clinical, and psychological characteristics previously associated with gambling problems. Given the cross-sectional design, the term “predictor” refers to statistical prediction and does not imply temporal precedence, causal influence, or independence from the clinical manifestation of GD.

## 3 Results

### 3.1 Characteristics of the sample

**Table 1** shows the characteristics of the HC and GD subsamples. Marked differences were observed in gender distribution: women represented 80.1% of the HC subsample, whereas men represented 83.5% of the GD subsample. Marital status also differed descriptively between groups. Most HC participants were married (86.0%), compared with 51.9% of participants in the GD subsample, whereas single (18.1%), separated/divorced (22.8%), and widowed (7.1%) participants represented larger proportions of the GD subsample. These differences should be considered when interpreting the classification performance and the generalizability of the model.

**Table 1:** Description for the variables collected for the GD and the HC subsamples.

	HC (n = 136)		GD (n = 745)	
	n	%	n	%
Gender				
Women	109	80.1%	123	16.5%
Men	27	19.9%	622	83.5%
Marital status				
Single	6	4.4%	135	18.1%
Married	117	86.0%	387	51.9%
Separated-divorced	9	6.6%	170	22.8%
Widowed	4	2.9%	53	7.1%
Education				
Primary	88	64.7%	501	67.2%
Secondary	23	16.9%	211	28.3%
University	25	18.4%	33	4.4%
Employment				
Unemployed	74	54.4%	280	37.6%
Retired	15	11.0%	217	29.1%
Employed	47	34.6%	248	33.3%
Social position				
High	9	6.6%	8	1.1%
Mean-high	9	6.6%	31	4.2%
Mean	18	13.2%	63	8.5%
Mean-low	82	60.3%	410	55.0%
Low	18	13.2%	233	31.3%
Origin				
Spain	125	91.9%	704	94.5%
Other	11	8.1%	41	5.5%
Chronological age	Mean	SD	Mean	SD
Age (years-old)	58.14	6.10	60.94	8.10
DERS	Mean	SD	Mean	SD
Non acceptance	10.80	3.34	17.04	4.53
Goals	10.88	2.95	13.03	3.02
Impulse	9.76	3.18	12.16	3.10
Awareness	14.90	3.39	17.80	3.44
Strategy	14.24	4.56	18.82	4.85
Clarity	8.89	2.85	11.17	2.68
Total	69.04	15.56	90.06	14.96
TCI-R	Mean	SD	Mean	SD
Novelty seeking	88.83	10.50	105.03	10.83
Harm avoidance	95.19	13.99	104.22	13.34
Reward depend.	102.21	13.12	98.03	12.46
Persistence	103.80	14.48	104.04	16.22
Self-directedness	145.79	20.13	128.86	17.71
Cooperativeness	137.96	15.94	130.92	12.71
Self-transcend.	62.05	12.58	65.17	12.53
SCL-90R	Mean	SD	Mean	SD
Somatization	0.88	0.56	1.12	0.89
Obsessive	0.85	0.56	1.23	0.89
Sensitivity	0.67	0.57	1.02	0.83
Depressive	1.01	0.63	1.62	0.98
Anxiety	0.68	0.47	1.05	0.85
Hostility	0.53	0.47	0.80	0.79
Phobic anxiety	0.28	0.32	0.55	0.74
Paranoia	0.59	0.46	0.96	0.83
Psychotic	0.40	0.42	0.94	0.79
GSI	0.71	0.45	1.12	0.74
PST	31.82	15.01	46.61	21.58
PSDI	1.81	0.45	2.00	0.66

HC, healthy control subsample; GD, gambling disorder subsample; SD, standard deviation.

Regarding educational level, primary education was the most common attainment in both groups, with smaller proportions of participants reporting secondary or university studies. Employment status included unemployed, retired, and employed individuals in both subsamples. Social position categories ranged from high to low, with most participants situated in the intermediate levels. The majority of participants in both groups were of Spanish origin. Descriptive statistics for the DERS, SCL-90-R, and TCI-R measures are also presented in **Table 1**.

**Table 2** summarizes the gambling-related, clinical, and psychological characteristics of participants in the GD subsample. Slot machines were the most frequently reported gambling activity, whereas other forms such as bingo, lotteries, casino games, sports betting and cards were less common. A minority of participants reported engagement in strategic forms of gambling or online gambling. Regarding substance use, tobacco consumption was the most prevalent, followed by alcohol use and, less frequently, other illicit substances. With respect to the course of the disorder, mean of age of onset of the GD was 51.4 years-old, and the duration of the problematic gambling 6.4 years. Regarding DSM-5 gambling disorder severity, most participants were classified in the higher severity ranges, with 42.1% endorsing 8–9 criteria (severe) and 35.2% endorsing 6–7 criteria (moderate). Smaller proportions met 4–5 criteria (mild; 14.8%) or presented subthreshold symptom levels (1–3 criteria; 8.0%).

**Table 2:** Description for the variables analyzed in the study among the GD subsample (N = 745).

Gambling activity	n	%
Slot-machines	580	77.85%
Bingo	72	9.66%
Lotteries	135	18.12%
Sport betting	20	2.68%
Casino	43	5.77%
Gambling rooms	28	3.76%
Cards	18	2.42%
Other	10	1.34%
Gambling preference	n	%
Strategic gambling	131	17.58%
Online gambling	34	4.56%
Substances use	n	%
Tobacco	338	45.37%
Alcohol	127	17.05%
Other illegal drugs	34	4.56%
Onset / progression of GD	Median	IQR
Age of onset of GD (yrs)	53.00	16.00
Duration of GD (yrs)	3.00	8.00
GRCS	Mean	SD
Expectancies	12.86	4.67
Illusion of control	7.97	3.64
Predictive control	15.17	5.80
Inability to stop	17.88	5.52
Interpretation bias	12.91	4.88
Total	66.81	20.48
UPPS-P	Mean	SD
Lack premeditation	23.09	4.87
Lack perseverance	21.74	4.18
Sensation seeking	24.12	6.10
Positive urgency	32.17	8.01
Negative urgency	31.78	5.59
Total	132.90	19.82
GD severity (DSM-5 criteria)	n	%
Subthreshold (1-3 criteria)	7	7.95%
Mild 4-5 (criteria)	13	14.77%
Moderate (6-7 criteria)	31	35.23%
Severe (8-9 criteria)	37	42.05%

GD, gambling disorder; SD, standard deviation; IQR, interquartile range.

### 3.2 Neural network-based prediction of the group classification (GD versus HC)

The neural network model developed to classify participants as belonging to the GD (N = 745) or HC (N = 136) group consisted of a single hidden layer with eight neurons. Its classification performance across the training, testing, holdout, and combined samples is presented in **Table 3**. Overall, the model showed high discriminative accuracy in all data partitions. In the training and testing sets, both Se and Sp reached high values, indicating that the model was able to correctly identify most patients with GD while also accurately classifying a large proportion of HC participants. This pattern was further supported by the high PPV, NPV, F1 scores, and balanced accuracy observed in these subsets. Performance remained high in the holdout subsample, where 75 of 78 participants with GD were correctly classified (Se = 96.2%) and 9 of 12 HC participants were correctly classified (Sp = 75.0%), resulting in an overall classification accuracy of 93.3%. The corresponding PPV and NPV were both 96.2% and 75.0%, respectively, while the F1 score was 96.2% and balanced accuracy was 85.6%. However, the relatively small number of HC participants in the holdout subsample (n = 12) warrants caution when interpreting the specificity and related performance estimates. The holdout results nevertheless indicate that the model retained good classification performance in previously unused data. When considering the full sample, the model achieved a high overall classification rate (96.6%), with sensitivity (97.7%) exceeding specificity (90.4%). PPV was 98.2%, NPV was 87.9%, the F1 score was 98.0%, and balanced accuracy was 94.1%, indicating that the high overall classification accuracy was not solely attributable to the unequal distribution of GD and HC participants.

**Table 3:** Contingency table with the classification of the model predicting the presence of GD.

		Predicted		Accuracy						
Set	Observed	HC	GD	Sp	Se	PPV	NPV	BA	F1	OP
Training	HC	78	6	92.86%	98.41%	98.64%	91.76%	95.63%	98.52%	97.52%
	GD	7	434							
	Overall %	16.2%	83.8%							
Testing	HC	36	4	90.00%	96.90%	98.21%	83.72%	93.45%	97.55%	95.86%
	GD	7	219							
	Overall %	16.2%	83.8%							
Holdout	HC	9	3	75.00%	96.15%	96.15%	75.00%	85.58%	96.15%	93.33%
	GD	3	75							
	Overall %	13.3%	86.7%							
Combined (including training data)	HC	123	13	90.44%	97.72%	98.25%	87.86%	94.08%	97.98%	96.59%
	GD	17	728							
	Overall %	15.9%	84.1%							

HC, healthy control subsample; GD, gambling disorder subsample; Sp, specificity; Se, sensitivity; PPV, positive predictive value; NPV, negative predictive value; BA, balanced accuracy; F1, F1 score; OP, overall performance.

The upper panel of **Figure 1** displays the normalized importance of the input variables in the classification model. These indices are calculated from the connection weights linking the input, hidden, and output layers of the network and reflect the relative contribution of each predictor to the model output. To facilitate interpretation, predictor importance values are normalized by assigning a value of 100% to the most influential predictor, with the remaining predictors expressed relative to this value. Accordingly, these indices should be interpreted as descriptive measures of relative influence within the fitted network rather than as estimates of independent statistical effects.

**Figure 1:**
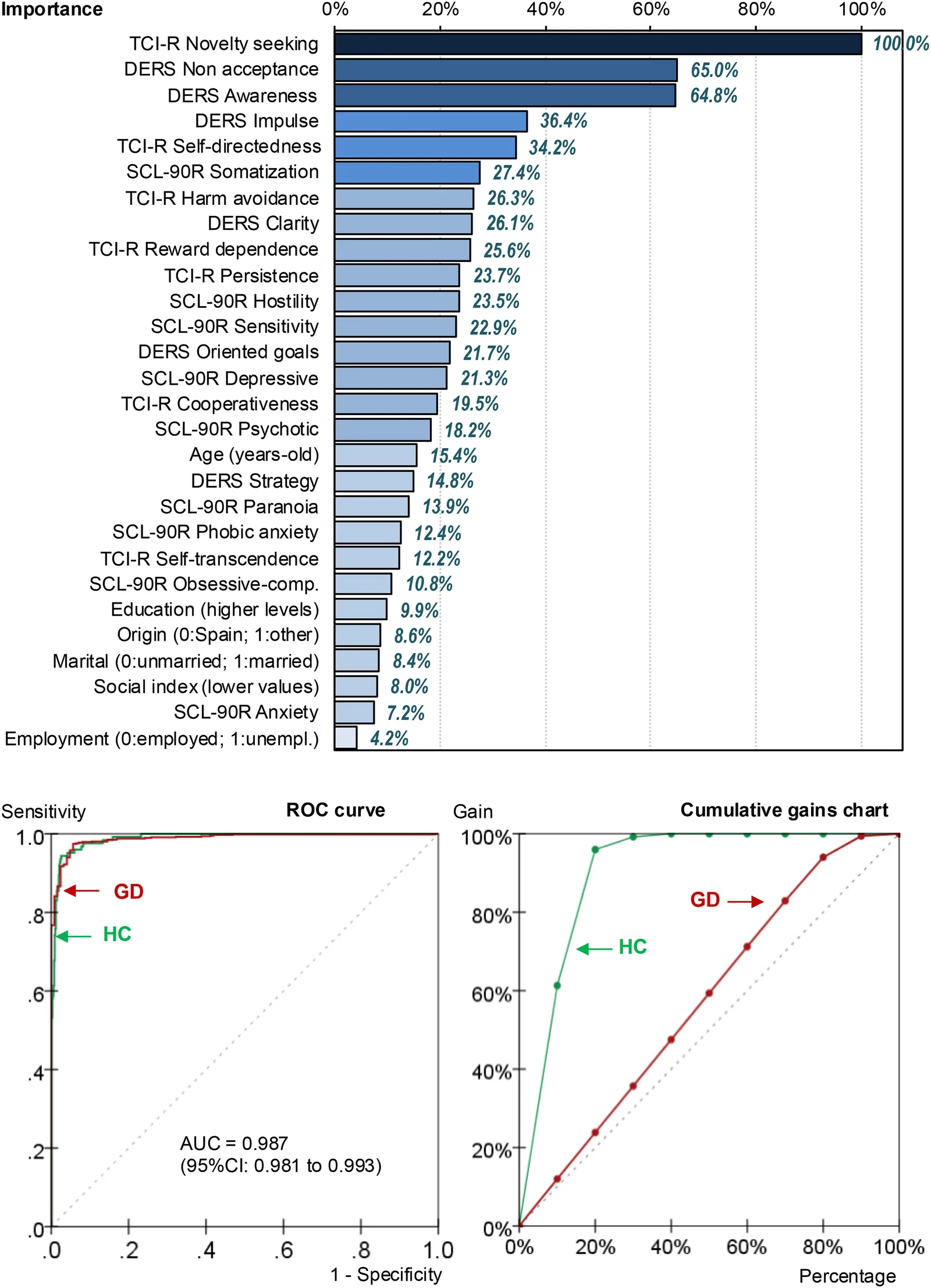
Importance of the predictors and ROC curve for the model predicting the presence of GD. Upper panel: bar chart with the importance (normalized values) of the input layer (independent variables). Lower panel: ROC curve and cumulative gains chart. AUC, area under the ROC curve. 95%CI: 95% confidence interval.

A temperament-personality trait, particularly novelty seeking, showed the highest relative importance, followed by several dimensions of emotion regulation difficulties (notably non-acceptance of emotions and lack of emotional awareness). Measures of general psychopathological symptoms and additional personality dimensions also contributed to the model, although with lower relative weights. Sociodemographic variables such as age, education, origin, marital status, and employment status showed comparatively smaller contributions to the classification function.

The lower left panel of **Figure 1** presents the ROC curve for the model in the complete analytical sample. The curve lies close to the upper-left corner of the plot, indicating excellent discriminative capacity across classification thresholds, with an AUC of 0.987 (95% confidence interval [95%CI]: 0.981 to 0.993). To further assess the model’s discriminative performance in previously unused data, an additional ROC analysis was conducted in the holdout subset, yielding an AUC of 0.890 (95%CI: 0.763 to 0.999), which indicates good discriminative capacity. The lower right panel shows the cumulative gains chart, which provides a complementary assessment of the model’s classification performance by showing the proportion of participants from each outcome category identified when individuals are ranked according to their predicted probabilities. The cumulative gains curve for HC showed a marked departure from the diagonal reference line, indicating that HC participants were concentrated efficiently within the corresponding ranked predictions. In contrast, the GD curve was closer to the diagonal. The asymmetry between the cumulative gains curves reflects the unequal prevalence of GD and HC participants in the sample and should not be interpreted as differential model performance across classes. These findings should be interpreted as complementary to the classification performance measures reported in **Table 3**, which showed high sensitivity and specificity at the model’s classification threshold.

Regarding the repeated random split validation procedure, mean holdout AUC across the 100 repetitions was 0.844 (SD = 0.046, CV = 0.054, range = 0.769-0.971), indicating consistently high discriminative capacity. Mean sensitivity remained very high and particularly stable (0.961, SD = 0.021, CV = 0.022, range = 0.896-0.989). Mean specificity was 0.732 (SD = 0.120, CV = 0.163, range = 0.467-0.933) and showed greater variability across partitions, reflecting the limited number of healthy controls available in the holdout subsets. Mean classification accuracy was 0.927 (SD = 0.030, CV = 0.032, range = 0.846-0.973), suggesting high stability across repetitions. Overall, the low CV values observed for AUC, sensitivity, and accuracy support the robustness and generalizability of the classification model across alternative sample partitions, whereas the comparatively higher variability of specificity is consistent with sampling fluctuations expected from the small holdout control samples.

**Table 4** presents the parameter estimates of the neural network model. Examination of the output-layer weights identified H(1:8) and H(1:6) as the hidden neurons with the strongest contributions to the GD = 1 output, whereas H(1:4) showed the strongest contribution to the GD = 0 output. Examination of the input-layer weights of these neurons was then used to characterize three model-derived patterns based on the predominant input variables contributing to each hidden neuron. However, these interpretations should be approached with caution, as the identified latent patterns emerged from the internal representation learned by the neural network and therefore constitute exploratory characterizations of the fitted model. Given that different parameterizations may yield equivalent predictive functions, the neuron-level patterns observed in the present study require replication and formal validation before they can be considered evidence of stable clinical subtypes.

**Table 4:** Parameter estimates for the model predicting the presence of GD.

		Hidden layer 1								Output layer	
	Predictor	H(1:1)	H(1:2)	H(1:3)	H(1:4)	H(1:5)	H(1:6)	H(1:7)	H(1:8)	0=HC	1=GD
Input Layer	(Bias)	0.850	-0.260	-0.757	-0.825	-0.369	1.436	-0.551	0.653		
	Marital (0:unmarried; 1:married)	-0.222	0.083	0.325	0.213	-0.158	-0.267	-0.177	-0.330		
	Education (higher levels)	0.407	0.269	0.240	0.286	0.168	0.169	0.099	-0.060		
	Employment (0:employed; 1:unempl.)	-0.016	0.079	0.121	-0.026	-0.254	0.118	-0.038	-0.260		
	Social index (lower values)	-0.431	0.273	0.210	-0.065	-0.102	0.012	-0.075	0.018		
	Origin (0:Spain; 1:other)	0.328	0.057	-0.230	0.026	0.156	0.030	0.084	-0.527		
	Age (years-old)	0.301	-0.328	0.527	0.007	0.423	0.180	-0.254	0.044		
	DERS Non acceptance	-0.057	-0.616	-0.033	-0.804	0.048	1.378	-0.962	0.401		
	DERS Oriented goals	0.208	-0.010	0.164	0.439	-0.421	0.570	0.087	0.214		
	DERS Impulse	-0.398	-0.071	0.521	0.331	-0.014	-0.223	-0.027	0.515		
	DERS Awareness	-0.005	-0.442	-0.416	-0.596	0.619	0.715	-0.289	0.023		
	DERS Strategy	-0.094	-0.269	0.449	-0.001	-0.511	0.520	-0.201	0.069		
	DERS Clarity	0.208	-0.705	0.603	0.076	-0.073	0.798	-0.160	0.521		
	SCL-90R Somatization	-0.471	0.387	0.588	0.050	-0.035	-0.778	-0.117	0.179		
	SCL-90R Obsessive-comp.	0.161	0.348	0.284	-0.298	0.107	-0.301	-0.013	0.046		
	SCL-90R Sensitivity	0.300	0.672	0.098	0.216	0.362	-0.219	0.576	0.069		
	SCL-90R Depressive	-0.247	-0.485	0.334	-0.180	0.093	0.398	-0.162	0.423		
	SCL-90R Anxiety	-0.078	0.090	-0.136	-0.079	-0.022	-0.358	-0.515	-0.238		
	SCL-90R Hostility	-0.088	-0.288	0.644	0.329	-0.367	-0.332	-0.082	0.139		
	SCL-90R Phobic anxiety	0.082	0.265	0.306	-0.131	0.245	0.318	-0.066	-0.348		
	SCL-90R Paranoia	0.328	0.318	-0.019	-0.233	-0.506	-0.004	0.249	0.284		
	SCL-90R Psychotic	-0.243	-0.508	0.235	-0.829	-0.339	0.018	0.269	-0.168		
	TCI-R Novelty seeking	0.415	-0.174	-0.574	-1.735	0.186	0.590	-1.078	1.422		
	TCI-R Harm avoidance	-0.080	-0.328	0.225	-0.470	0.039	-0.701	0.124	0.227		
	TCI-R Reward dependence	0.414	0.175	0.244	0.053	0.106	0.196	0.299	-0.858		
	TCI-R Persistence	-0.279	0.122	-0.119	-0.312	-0.163	0.665	0.037	-0.521		
	TCI-R Self-directedness	0.453	-0.596	0.228	0.795	0.517	-0.157	-0.549	-0.754		
	TCI-R Cooperativeness	0.372	-0.653	-0.297	0.053	-0.224	0.131	-0.082	-0.492		
	TCI-R Self-transcendence	0.223	0.002	0.012	0.013	-0.321	-0.175	-0.416	-0.214		
Hidden Layer	(Bias)									-2.029	1.786
	H(1:1)									-0.081	0.010
	H(1:2)									0.312	-1.261
	H(1:3)									0.568	-0.488
	H(1:4)									1.531	-1.524
	H(1:5)									-0.950	0.187
	H(1:6)									-0.900	0.905
	H(1:7)									0.336	-0.417
	H(1:8)									-0.619	0.935

HC, healthy control subsample; GD, gambling disorder subsample.

The weights linking the input variables to neuron H(1:8) suggests a pattern characterized by very high novelty seeking, greater depressive symptomatology, and elevated difficulties in emotion regulation, particularly in domains related to non-acceptance of emotional responses, impaired impulse control and limited emotional clarity. This neuron also showed negative associations with personality traits such as self-directedness, cooperativeness, and reward dependence, suggesting a combination of impulsive–exploratory temperament, emotional dysregulation, and lower adaptive personality functioning. This pattern suggests that neuron H(1:8) reflects an “*impulsive–exploratory dysregulation”* pattern.

Neuron H(1:6) was strongly linked to emotion regulation difficulties (in all the domains, except in impulse control difficulties and reduced emotional clarity), together with elevated depressive symptoms and phobic anxiety. In contrast, it showed negative associations with harm avoidance, suggesting a tendency toward reduced threat sensitivity alongside poor emotional control. This configuration may reflect a pattern marked by emotion-driven dysregulation and diminished inhibitory control, which was associated with GD classification in the present model. Neuron H(1:6) reflects an “*emotion-driven dysregulation”* pattern.

Regarding neuron H(1:4), which was the most strongly associated with the absence of GD (GD = 0), it suggests a comparatively distinct pattern. It shows negative associations with psychopathological indicators such as depressive symptoms, somatization, psychoticism, and difficulties in non-acceptance and awareness of emotions. In contrast, positive associations with adaptive personality traits, particularly self-directedness, indicate goal-oriented functioning and effective self-regulation. Strong negative weights for novelty seeking further suggest lower impulsivity and exploratory tendencies. Overall, this configuration reflects a pattern characterized by emotional stability, adaptive personality functioning, and lower levels of characteristics contributing to GD classification in the present model. Neuron H(1:4) reflects an “*adaptive–resilient”* pattern.

### 3.3 Neural network–based prediction of GD severity

The neural network model was used to predict the severity of GD among the GD group (N = 745), operationalized as the number of DSM-5 criteria met by each patient. The model included a hidden layer with six neurons. The upper panel of **Figure 2** displays the input variables ordered by their relative importance. Psychological distress, as measured by the SCL-90R Global Severity Index (GSI), emerged as the strongest contributing variable, followed by novelty seeking (TCI-R), cognitive biases related to gambling (GRCS) and self-directedness (TCI-R). In contrast, sociodemographic variables, including gender, education, marital status, and socioeconomic index, as well as substance use, showed lower relative importance in the model.

**Figure 2:**
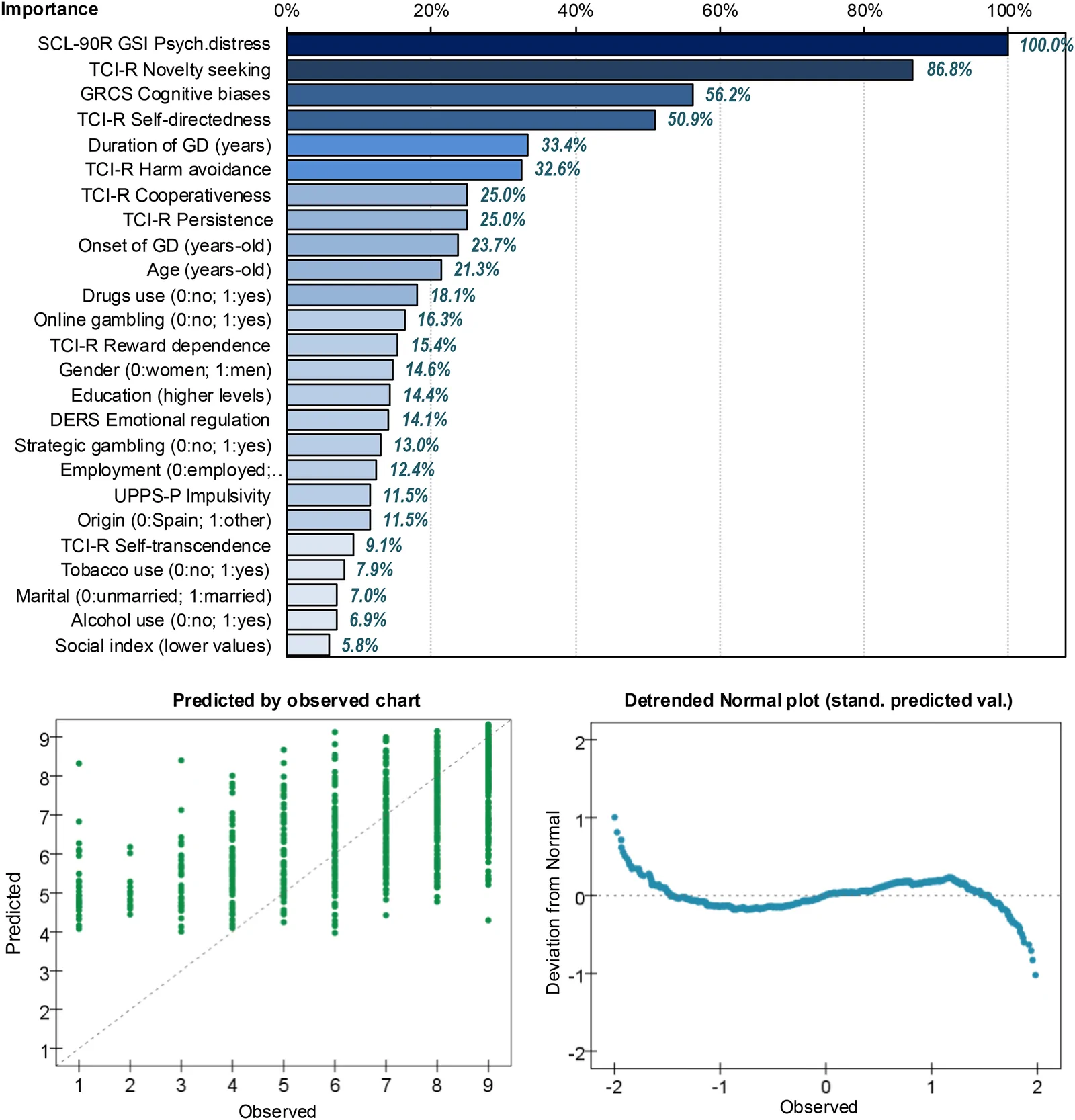
Importance of the predictors for the models predicting the severity of the GD. Upper panel: bar chart with the importance (normalized values) of the input layer (independent variables). Lower panel: predicted by observed chart and detrended normal plot (standardized values).

The lower left panel shows the predicted-by-observed plot. Most predicted values align closely with the observed DSM-5 criteria, suggesting that the model captures the general pattern of GD severity in the sample. Some dispersion is observed at the extremes, reflecting variability that may not be fully explained by the input variables. The lower right panel presents the detrended normal plot, which illustrates deviations of predicted values from a standardized normal distribution. The curve remains near zero across most of the range, indicating the absence of systematic prediction bias, with only minor deviations for extreme scores. The model explained 35.0% of the variance in the outcome (adjusted R² = 0.350), and predictive accuracy was acceptable (RMSE = 1.78 and a MAE = 1.38). Internal validation based on 100 repeated random splits showed consistent model performance. Across repetitions, the mean adjusted R² was 0.320 (SD = 0.096; range = 0.098-0.536), while the mean RMSE and MAE were 1.765 (SD = 0.179; range = 1.384-2.273) and 1.394 (SD = 0.139; range = 1.093-1.765), respectively, indicating moderate predictive accuracy and acceptable stability across data partitions.

**Table 5** contains the parameters of the neural network model predicting GD severity. These results revealed that three hidden neurons were particularly influential: H(1:3) was associated with higher predicted GD severity, whereas H(1:4) and H(1:5) were associated with lower predicted severity.

**Table 5:** Parameter estimates for the model predicting the severity of the GD (DSM-5 criteria).

		Hidden layer						Output layer
Predictor		H(1:1)	H(1:2)	H(1:3)	H(1:4)	H(1:5)	H(1:6)	GD-severity
Input Layer	*(Bias)*	*0.291*	*0.405*	*-0.011*	*0.088*	*-0.433*	*-0.102*	
	Gender (0:women; 1:men)	-0.261	-0.075	0.387	0.092	-0.403	0.024	
	Marital (0:unmarried; 1:married)	-0.398	0.044	-0.008	0.301	-0.385	0.271	
	Education (higher levels)	-0.467	-0.271	0.155	-0.175	0.218	-0.395	
	Employment (0:employed; 1:unemployed)	0.220	0.350	-0.303	0.374	0.184	0.313	
	Social index (lower values)	0.055	0.120	-0.182	-0.046	0.005	0.280	
	Origin (0:Spain; 1:other)	0.380	0.410	0.396	0.153	0.102	-0.131	
	Age (years-old)	-0.523	-0.240	0.191	-0.082	0.348	-0.037	
	Onset of GD (years-old)	-0.348	-0.344	-0.305	0.461	-0.323	-0.163	
	Duration of GD (years)	0.437	-0.199	0.305	0.020	-0.121	-0.068	
	Strategic gambling (0:no; 1:yes)	0.201	0.004	0.435	-0.219	-0.071	-0.371	
	Online gambling (0:no; 1:yes)	-0.325	-0.275	-0.124	-0.438	-0.036	0.021	
	Tobacco use (0:no; 1:yes)	-0.035	0.209	0.420	-0.062	0.192	0.571	
	Alcohol use (0:no; 1:yes)	-0.109	0.079	0.272	0.341	0.051	0.187	
	Drugs use (0:no; 1:yes)	-0.450	0.145	-0.033	0.227	-0.349	0.054	
	SCL-90R GSI	0.382	-0.193	0.518	-0.470	-0.778	0.700	
	UPPS-P Impulsivity	-0.221	-0.005	0.200	0.205	-0.098	-0.010	
	DERS Emotional regulation	0.080	0.537	0.280	0.081	-0.073	-0.414	
	GRCS Cognitive biases	0.244	-0.432	0.485	-0.310	0.099	0.287	
	TCI-R Novelty seeking	-0.093	-0.280	0.084	-0.367	-0.596	0.557	
	TCI-R Harm avoidance	0.559	0.174	0.298	-0.138	-0.032	-0.271	
	TCI-R Reward dependence	0.385	0.308	-0.089	0.096	-0.245	-0.166	
	TCI-R Persistence	0.258	-0.089	0.427	-0.089	0.488	0.290	
	TCI-R Self-directedness	-0.378	-0.040	-0.217	0.419	0.153	-0.075	
	TCI-R Cooperativeness	-0.339	-0.053	0.288	0.588	-0.049	0.287	
	TCI-R Self-transcendence	0.075	0.298	0.223	0.008	0.150	0.117	
Hidden Layer	*(Bias)*							*0.041*
	H(1:1)							0.197
	H(1:2)							-0.079
	H(1:3)							0.212
	H(1:4)							-0.284
	H(1:5)							-0.316
	H(1:6)							0.154

GD, gambling disorder. GD, severity measured as the total number of DSM-5 criteria for the disorder.

Examination of the input weights for H(1:3) indicated that this neuron was primarily activated by being male, tobacco use, strategic forms of gambling, higher global psychopathology distress (SCL-90R GSI), cognitive distortions related to gambling (GRCS), and higher persistence (TCI-R). Thus, H(1:3) captures a pattern that can be described as “*higher-severity*” pattern.

In contrast, neuron H(1:4), associated with lower predicted severity. was strongly activated by being married, later onset of gambling problems, higher self-directedness and cooperativeness, moderate alcohol use, and lower online gambling and psychological symptoms. This pattern represents a distinct pattern associated with lower predicted severity within the GD subsample, characterized by psychosocial stability and self-regulation. It can be described as “*psychosocially stable lower-severity*” pattern.

Neuron H(1:5), also associated with lower predicted GD severity, was primarily influenced by lower general psychopathology, lower novelty seeking, female gender, and higher persistence, reflecting a pattern associated with lower predicted severity and adaptive traits. It can be described as “*adaptive lower-severity*” pattern.

### 3.4 Logistic regression–based prediction of the group classification (GD versus HC)

To assess the predictive performance of the first neural-network approach against a conventional statistical model, a binary logistic regression was fitted to predict GD classification (GD [N = 745] vs. HC [N = 136]) using the same set of predictors included in the MLP. Both models were fitted and evaluated using the full sample, ensuring that the comparison was based on the same participants and predictor set. The results are presented in **Table 6**. For ease of comparison with the MLP results, predictors in this table are presented as descriptive summaries of the regression models. The ordering of variables is intended solely to facilitate comparison between regression coefficients and the normalized importance indices produced by the neural network and should not be interpreted as a hierarchy of substantive predictor importance. In particular, statistical significance reflects the evidence for an adjusted association within the regression model, whereas normalized importance indices quantify the relative contribution of predictors to the neural network output. Moreover, because several predictors are conceptually related and were entered simultaneously into the models, some adjusted regression coefficients differed in direction from the corresponding univariate group comparisons. These coefficients should therefore be interpreted as conditional effects estimated after adjustment for the remaining predictors rather than as direct reflections of bivariate associations. Consistent with this interpretation, supplementary collinearity diagnostics did not indicate severe dependencies among predictors (variance inflation factor [VIF] values into the range 1.07 to 6.16), although suppression effects may still arise when multiple conceptually related variables are included simultaneously in multivariable models.

**Table 6:** Logistic regression model distinguishing HC and GD subsamples.

	*B*	*SE*	*p-value*	*OR*
TCI-R Novelty seeking	0.1542	0.0207	1.087E-13	1.1667
DERS Non acceptance	0.5101	0.0833	8.999E-10	1.6655
DERS Awareness	0.3313	0.0599	3.266E-08	1.3928
Marital status (0:unmarried; 1:married)	-1.9901	0.5058	8.336E-05	0.1367
SCL-90R Sensitivity	-2.5352	0.6461	8.703E-05	0.0792
Age (years-old)	0.1201	0.0341	4.185E-04	1.1277
SCL-90R Psychotic	1.9877	0.5969	8.678E-04	7.2984
SCL-90R Somatization	-1.1714	0.4694	1.258E-02	0.3099
Origin (0:Spain; 1:other)	-1.6462	0.7114	2.067E-02	0.1928
SCL-90R Depressive	1.2374	0.5384	2.155E-02	3.4465
DERS Oriented goals	-0.2038	0.0968	3.525E-02	0.8156
Employment (0:employed; 1:unemploy.)	-0.9037	0.4572	4.810E-02	0.4051
Education (higher levels)	-0.7486	0.3877	5.350E-02	0.4730
DERS Impulse	-0.1456	0.0966	1.316E-01	0.8645
TCI-R Self-directedness	-0.0217	0.0155	1.603E-01	0.9785
TCI-R Self-transcendence	-0.0213	0.0171	2.138E-01	0.9789
TCI-R Cooperativeness	0.0258	0.0218	2.364E-01	1.0262
TCI-R Harm avoidance	0.0151	0.0153	3.230E-01	1.0152
TCI-R Persistence	0.0150	0.0159	3.469E-01	1.0151
SCL-90R Paranoia	0.4104	0.5258	4.351E-01	1.5075
DERS Clarity	-0.0584	0.0994	5.568E-01	0.9433
DERS Strategy	0.0484	0.0876	5.808E-01	1.0495
SCL-90R Hostility	-0.1693	0.5423	7.549E-01	0.8442
SCL-90R Obsessive-compulsive	-0.1190	0.5668	8.337E-01	0.8878
SCL-90R Anxiety	0.1241	0.7394	8.668E-01	1.1321
TCI-R Reward dependence	-0.0029	0.0193	8.820E-01	0.9971
Social index (lower values)	-0.0082	0.2959	9.780E-01	0.9919
SCL-90R Phobic anxiety	-0.0092	0.6693	9.891E-01	0.9909
*Global discrimination indexes*				
Classification table:				
	Predicted			
Observed	HC	GD		
HC	113	23		
GD	26	719		
Overall %	15.78%	84.22%		
Accuracy:	Sp	83.09%		
	Se	96.51%		
	PPV	96.90%		
	NPV	81.29%		
	BA	89.80%		
	F1	96.70%		
	OP	94.44%		

HC, healthy control subsample; GD, gambling disorder subsample.

Dependent variable coded as GD = 1 and HC = 0.

SE, standard error. OR, odds ratio; Sp, specificity; Se, sensitivity; PPV, positive predictive value; NPV, negative predictive value; BA, balanced accuracy; F1, F1 score; OP, overall performance.

The logistic regression showed high discriminative performance, with an AUC of 0.977, compared with an AUC of 0.987 for the MLP. Although the difference in AUC was relatively small, the MLP showed consistently better performance across all classification indices at the selected classification threshold. The MLP yielded an overall accuracy of 96.59%, sensitivity of 97.72%, specificity of 90.44%, balanced accuracy of 94.08%, PPV of 98.25%, NPV of 87.86%, and an F1 score of 97.98%, compared with 94.44%, 96.51%, 83.09%, 89.80%, 96.90%, 81.29%, and 96.70%, respectively, for the logistic regression model (**Table 6**). The largest difference was observed for specificity: the MLP correctly classified 123 of the 136 HC participants, whereas the logistic regression correctly classified 113 of 136. Regarding calibration metrics, analyses indicated good agreement between observed and predicted probabilities for both models (**Figure 3**). Visual inspection of the calibration plots showed that predictions closely followed the line of perfect calibration across the range of estimated probabilities, although the MLP exhibited slightly closer agreement with the observed proportions. Consistent with these observations, The MLP yielded a lower Brier score than the logistic regression model (0.029 vs. 0.038), suggesting slightly better overall probabilistic performance.

**Figure 3:**
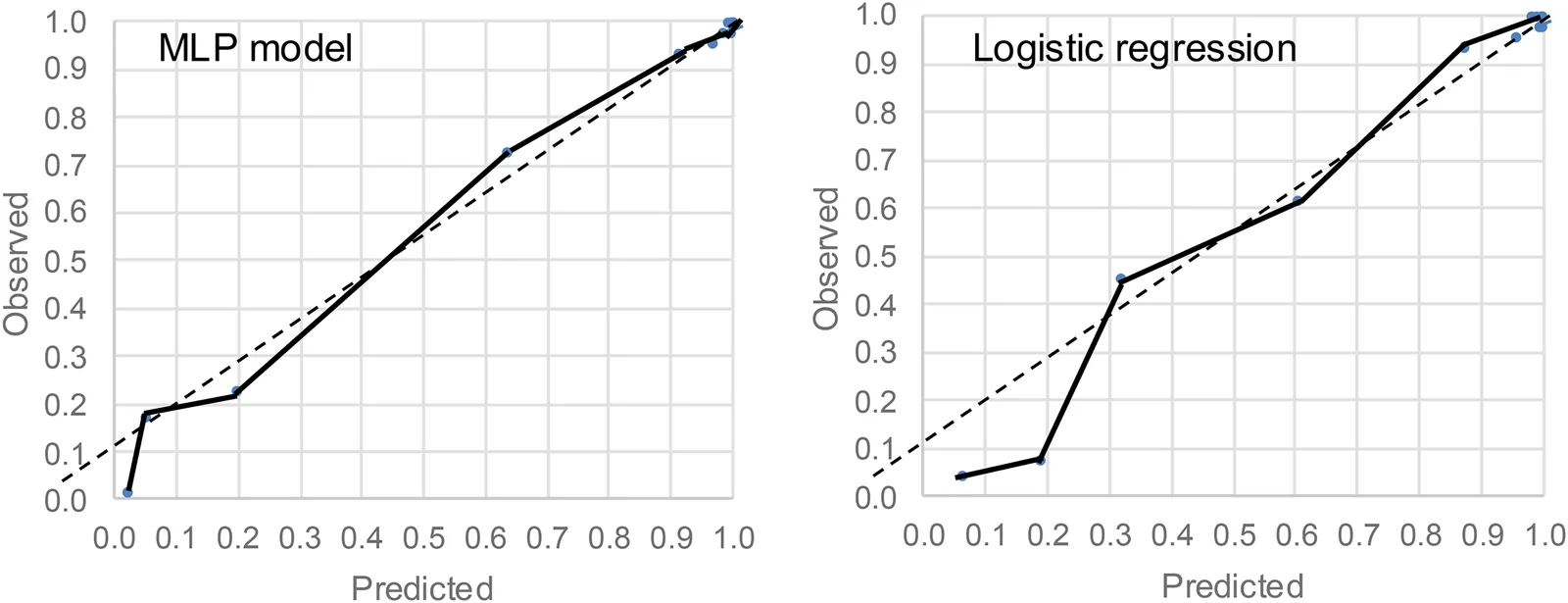
Calibration plots for the MLP and the logistic regression. The diagonal dashed line represents perfect calibration (observed probabilities equal predicted probabilities). Points closer to the diagonal indicate better agreement between predicted and observed probabilities.

The comparison of predictor contributions revealed further differences between the two modeling approaches. In the logistic regression, 12 predictors showed statistically significant independent associations with GD at the conventional *p* <.05 threshold. The strongest predictors were TCI-R novelty seeking, DERS non-acceptance, and DERS awareness, followed by marital status, SCL-90-R sensitivity, age, SCL-90-R psychoticism, SCL-90-R somatization, origin, SCL-90-R depression, DERS oriented goals, and unemployment. The remaining predictors did not reach statistical significance. Notably, the three predictors with the highest relative importance in the MLP (TCI-R novelty seeking, DERS non-acceptance of emotions, and DERS lack of awareness) were also the three strongest predictors in the logistic regression. However, several predictors that were not statistically significant in logistic regression showed relevant contributions to the MLP. For example, DERS impulse control and TCI-R self-directedness had relative importance values of 36.4% and 34.2%, respectively, in the MLP, despite showing non-significant associations in the logistic regression (*p* = 0.132 and *p* = 0.160, respectively). Similarly, TCI-R harm avoidance, DERS clarity, TCI-R reward dependence, TCI-R persistence, and SCL-90-R hostility contributed to the neural-network model despite not showing statistically significant independent effects in the logistic regression. This is particularly relevant because the neural network does not evaluate each predictor only through an independent global effect; rather, predictors contribute jointly through combinations of weighted connections across hidden neurons. This suggests that some predictors contribute to GD classification primarily through interactions or nonlinear combinations with other variables rather than through a strong independent linear association with the outcome.

This distinction is particularly reflected in the descriptive interpretation of the hidden neurons. For example, neuron H(1:8) was characterized by high novelty seeking together with difficulties in emotional non-acceptance, impulse control, and emotional clarity, combined with lower self-directedness, cooperativeness, and reward dependence. Thus, variables that were not individually significant in the logistic regression may nevertheless contribute meaningfully to a multivariate configuration represented by a specific hidden neuron. Similarly, H(1:6) combined emotion-regulation difficulties with depressive symptoms, phobic anxiety, and lower harm avoidance, whereas H(1:4), which was most strongly associated with the absence of GD, represented a comparatively adaptive configuration characterized by lower psychopathological symptoms and emotion-regulation difficulties, higher self-directedness, and lower novelty seeking.

### 3.5 Ordinal regression–based prediction of GD severity

To further evaluate the predictive performance of the MLP, an ordinal regression model with a cumulative logit link function was fitted to predict GD severity in the clinical subsample (N = 745). Severity was operationalized as the number of DSM-5 criteria fulfilled. Given the ordered nature of the outcome and its bounded range, ordinal regression was considered a more appropriate conventional statistical approach for comparison than models assuming an unbounded count distribution. The ordinal regression model and the MLP were fitted and evaluated using the full clinical sample and the same set of predictors. Parameter estimates are presented in **Table 7**, with input variables ordered according to their relative contribution to the ordinal regression model based on their statistical significance. As in the logistic regression analysis, predictors are presented as descriptive summaries of the model to facilitate comparison with the normalized importance indices derived from the neural network. Collinearity diagnostics did not indicate problematic multicollinearity among predictors (all VIFs < 2.4), although suppression effects may still arise when multiple related variables are estimated simultaneously in a multivariable model.

**Table 7:** Ordinal regression model predicting the GD severity.

Predictor	*B*	*SE*	*p-value*	*OR*
SCL-90R GSI	0.9074	0.1260	6.005E-13	2.4780
GRCS Cognitive biases	0.0222	0.0040	4.076E-08	1.0225
TCI-R Novelty seeking	0.0311	0.0073	1.815E-05	1.0316
Age (years-old)	-0.0438	0.0124	4.000E-04	0.9572
Duration of GD (years)	0.0169	0.0048	5.000E-04	1.0170
TCI-R Self-directedness	-0.0155	0.0059	8.400E-03	0.9846
Drugs use (0:no; 1:yes)	0.2140	0.1327	1.068E-01	1.2386
Gender (0:women; 1:men)	0.2905	0.1949	1.360E-01	1.3371
TCI-R Harm avoidance	0.0091	0.0067	1.723E-01	1.0092
Alcohol use (0:no; 1:yes)	-0.1371	0.1210	2.572E-01	0.8719
Strategic gambling (0:no; 1:yes)	0.2286	0.2072	2.699E-01	1.2568
Marital status (0:unmarried; 1:married)	-0.1428	0.1375	2.989E-01	0.8669
UPPS-P Impulsivity	-0.0045	0.0044	3.000E-01	0.9955
Origin (0:Spain; 1:other)	0.2824	0.3102	3.626E-01	1.3263
DERS Emotional regulation	-0.0048	0.0055	3.840E-01	0.9952
Tobacco use (0:no; 1:yes)	0.0047	0.0061	4.388E-01	1.0047
TCI-R Self-transcendence	-0.0052	0.0072	4.720E-01	0.9949
Education (higher levels)	-0.0956	0.1453	5.108E-01	0.9089
TCI-R Reward dependence	0.0042	0.0069	5.403E-01	1.0042
Employment (0:employed; 1:unemployed)	-0.0957	0.1705	5.746E-01	0.9088
Social index (lower values)	-0.0473	0.1047	6.515E-01	0.9538
Online gambling (0:no; 1:yes)	-0.1133	0.3699	7.594E-01	0.8929
TCI-R Cooperativeness	-0.0014	0.0074	8.486E-01	0.9986
Onset of GD (years-old)	-0.0011	0.0067	8.645E-01	0.9989
TCI-R Persistence	-0.0004	0.0052	9.387E-01	0.9996

GD, gambling disorder. GD-severity measured as the total number of DSM-5 criteria for the disorder.

SE, standard error; OR, odds ratio.

The model significantly improved fit compared with the intercept-only model, χ²(25) = 332.82, *p* < 0.001. Goodness-of-fit statistics indicated an adequate fit to the data (Pearson χ² = 5668.97, *p* = 0.992; Deviance χ² = 2656.79, *p* = 1.000), and the proportional odds assumption was met according to the test of parallel lines, χ²(175) = 192.20, *p* = 0.177. The model explained a substantial proportion of variance in GD severity (Nagelkerke pseudo-R² = 0.367).

The MLP yielded an RMSE of 1.776, compared with 1.924 for the ordinal regression model, whereas MAE values were nearly identical (1.380 and 1.370, respectively). Likewise, the correlation between observed and predicted severity scores was comparable across models (r = 0.572 for the MLP and r = 0.591 for the ordinal regression model). These results indicate broadly similar predictive performance, although this comparison should be interpreted as an in-sample benchmark rather than a direct assessment of out-of-sample generalizability. For the MLP, the observed correlation corresponds to approximately 33% of the variance in GD severity (r² = 0.327), indicating that the model captured a meaningful proportion of the overall severity pattern while leaving substantial unexplained variability. The ordinal model produced a similar result (r = 0.591; r² = 0.349), accounting for approximately 35% of the variance in severity. Thus, both modeling approaches showed broadly comparable predictive performance, with only a small difference in the proportion of explained variance.

The contribution of individual predictors also differed between the two approaches. In the ordinal regression model, six predictors showed statistically significant associations with GD severity (p < 0.05): SCL-90-R GSI, GRCS cognitive biases, TCI-R novelty seeking, chronological age, duration of GD, and TCI-R self-directedness. The remaining predictors did not reach conventional significance levels. Nevertheless, there was substantial convergence between the ordinal regression and MLP results. SCL-90-R GSI showed the highest relative importance in the MLP (100.0%) and was also the strongest predictor in the ordinal model. TCI-R novelty seeking and GRCS cognitive biases ranked second and third in the MLP, with relative importance values of 86.8% and 56.2%, respectively, and were likewise among the strongest predictors in the ordinal regression model. TCI-R self-directedness ranked fourth in the MLP (50.9%) and also showed a significant association with GD severity in the ordinal model (p = 0.008).

Some discrepancies were also observed between approaches. Several variables that did not reach statistical significance in the ordinal regression nevertheless contributed to the predictive performance of the MLP. For example, TCI-R harm avoidance showed a relative importance of 32.6% in the MLP despite a non-significant ordinal regression coefficient (p = 0.172). Similarly, TCI-R cooperativeness and TCI-R persistence each showed a relative importance of 25.0% in the MLP, whereas their corresponding ordinal regression coefficients were not statistically significant.

Overall, the comparison between ordinal regression and MLP suggests substantial agreement regarding the main predictors of GD severity. Rather than identifying a completely different set of predictors, the neural network largely converged with the ordinal model on the most relevant variables while potentially capturing additional predictive information arising from complex multivariate relationships among predictors.

## 4 Discussion

These findings provide insights into sociodemographic, psychological, and personality variables associated with the presence of GD in older adults aged 50 to 85 years, as well as with the severity of the disorder. The model developed to discriminate between GD and HC highlighted the prominent role of personality, impulsivity, and emotion regulation variables. The model predicting GD severity evidenced the relevance of higher psychological distress, impulsive–exploratory personality traits, and gambling-related cognitive distortions. By integrating multidimensional assessment with predictive modeling, this study seeks to advance understanding of both vulnerability and resilience in later-life GD, and to provide clinically relevant knowledge to inform assessment, case formulation, and personalized intervention strategies in this growing and often overlooked population.

We acknowledge that the variables identified by the neural network represent statistical features contributing to classification rather than causal determinants of GD. Given the cross-sectional design, temporal and causal relationships cannot be established. Thus, associations involving psychological distress or gambling-related cognitive distortions may reflect bidirectional relationships or consequences of gambling involvement rather than pre-existing risk factors.

### 4.1 Discriminating between GD versus HC

The neural network model obtained excellent discriminative capacity in distinguishing individuals with GD from HC, with very high overall accuracy. The consistently higher Se than Sp indicates that the model was particularly effective at correctly identifying individuals with GD. This pattern is especially valuable in assessment contexts, where minimizing false negatives is often a priority. The identification of patterns associated with GD may be particularly relevant in older adults, a subpopulation in which identifying for problematic gambling may be less routinely considered. In this age group, clinical assessments often place substantial emphasis on physical health (68), with a strong focus on cardiometabolic indicators, as well as the detection of significant cognitive deficits. Consequently, behavioral conditions such as GD may receive comparatively less systematic attention, which could contribute to their underrecognition in some clinical settings.

A central finding is the greater relevance of personality traits and emotion regulation difficulties relative to sociodemographic variables in the classification process. In particular, the relevance of novelty seeking supports theoretical models that conceptualize GD as a behavioral addiction involving an impulsive–exploratory temperament. Classical personality-based frameworks, such as Cloninger’s psychobiological model (69), have consistently linked high novelty seeking to dopaminergic reward sensitivity, behavioral disinhibition, and a preference for immediate reinforcement. Consistent with this view, impulsive action and impulsive choice have been repeatedly associated with both substance-related and behavioral addictions, including gambling disorder (70). These perspectives are further echoed in addiction models emphasizing impulsivity and sensation seeking as characteristics associated with engagement with high-stimulation activities such as gambling (71). More recent models have expanded this view by integrating neurocognitive and motivational mechanisms, including dual-process frameworks that describe an imbalance between heightened reward-driven, impulsive systems and comparatively weaker reflective or control systems (72). Within contemporary accounts of GD, such as recent developments based on incentive sensitization theory (73) and transdiagnostic models of addictive behaviors (74), gambling environments are understood to provide potent and unpredictable reward cues that may be particularly salient for individuals with strong exploratory tendencies In the present sample of adults aged 50–85, the relevance of novelty seeking suggests that this trait may remain associated with GD in older adults, although longitudinal research would be needed to determine its role in the development or maintenance of gambling-related problems.

In parallel, the relevance of emotion regulation difficulties (particularly non-acceptance of emotional responses and lack of emotional awareness) aligns with modern integrative transdiagnostic frameworks that highlight affective dysregulation as an important process associated with GD (75–77), whereby gambling may serve both as a source of stimulation and as a maladaptive strategy for managing emotional states (78). In this context, gambling may function as a means of escaping, suppressing, or modulating poorly understood or poorly tolerated emotions, which is consistent with the high levels of affective symptomatology typically observed in individuals with GD. Together, these findings are compatible with lifespan-oriented conceptualizations of GD that emphasize stable personality dispositions and enduring regulatory processes rather than age-specific sociodemographic determinants. Previous studies have similarly reported that demographic indicators show smaller effect sizes in relation to GD (6).

The hidden neurons identified by the neural network may capture complex multivariate relationships among predictors that are not readily detectable using conventional statistical approaches. Nevertheless, the interpretation of these latent activation patterns remains speculative and should be considered hypothesis-generating rather than definitive evidence of distinct underlying clinical profiles. First, the hidden layer analysis further revealed psychologically meaningful patterns. The “*impulsive–exploratory dysregulation*” pattern was characterized by a combination of temperamental impulsivity, deficits in self-regulation, and personality functioning, which may be relevant to problematic gambling behaviors. The presence of this pattern in a sample of adults aged 50 to 85 years is not consistent with previous findings suggesting that impulsive and exploratory tendencies diminish uniformly across older adults (23). Rather, it suggests that core dispositional vulnerabilities may persist across the lifespan and may interact with age-related contextual conditions (10). Older adulthood is often accompanied by changes in daily routines, social roles, and reinforcement structures (such as retirement, increased leisure time, social isolation, or bereavement) that may influence reward-seeking behaviors or the opportunities for impulse control. In parallel, age-related neurobiological changes affecting frontostriatal circuits involved in reward processing and cognitive control (79, 80) may interact with pre-existing individual differences in impulsivity, potentially being relevant to dysregulated gambling behaviors. Additionally, the higher prevalence of pharmacological treatments in this age group, including medications with dopaminergic or activating properties, may be relevant in some vulnerable individuals to changes in reward-driven behaviors. Taken together, these findings are compatible with the possibility that later-life GD may, in part, reflect the convergence of enduring personality dispositions with age-specific contextual, neurobiological, and treatment-related factors, rather than a qualitatively distinct or *de novo* form of the disorder.

A second pattern, labeled “*emotion-driven dysregulation*” may reflect individuals who are not necessarily driven by intense sensation seeking but who may use gambling as a strategy to cope with intense or diffuse emotional states. In the context of adults aged 50 to 85 years, this pattern may be relevant to age-related emotional and psychosocial challenges (81) rather than primarily reflecting trait-like impulsive tendencies. Older adulthood is often characterized by cumulative exposure to loss, health-related stressors, and changes in interpersonal roles, which may increase emotional burden while simultaneously narrowing available coping resources. Although emotional regulation is frequently described as improving with age, this advantage may be less pronounced or may erode under conditions of chronic stress, loneliness, or subclinical affective symptoms (82). In such contexts, gambling could potentially function as an accessible and socially sanctioned activity that provides temporary emotional relief, distraction, or a sense of control, rather than high levels of stimulation (83, 84). This pattern may be consistent with a form of GD in older adults that is more closely related to affective vulnerability and emotion-focused coping within the aging process, highlighting heterogeneity in the psychological patterns associated with GD in older adults.

The coexistence of these patterns associated with the presence of GD suggests that in older adults this addiction may be characterized by multiple, partially overlapping vulnerability trajectories. Some individuals may reach older adulthood with long-standing temperamental and self-regulatory characteristics that remain relevant to reward-seeking behavior, whereas others may experience gambling-related problems in the context of age-specific emotional challenges, cumulative stressors, or changing motivational priorities. Importantly, these patterns are better understood as non–mutually exclusive, as impulsive predispositions and emotion-driven coping may converge over time, particularly in the presence of age-related changes in cognitive flexibility, stress reactivity, or environmental contingencies. This heterogeneity supports the potential value of a lifespan perspective in understanding GD, whereby later-life gambling problems can reflect both the persistence of earlier characteristics and the interaction of those vulnerabilities with developmental, psychological, and contextual processes characteristic of aging.

In contrast, the neuron most strongly associated with the absence of GD described an “*adaptive–resilient*” pattern, characterized by adaptive personality functioning and emotional competencies. Rather than indicating active protective factors, this pattern may reflect psychological characteristics associated with lower likelihood of GD in the present sample. Within adults aged 50 to 85 years, this pattern may reflect the consolidation of psychological resources accumulated across the lifespan (85), such as greater emotional clarity, acceptance, and the capacity to regulate behavior in accordance with long-term goals rather than immediate rewards. Older adulthood is often accompanied by shifts in motivational priorities toward emotional balance and meaningful engagement (86), which may be relevant to the lower salience of gambling as a source of either stimulation or emotional escape. Moreover, accumulated life experience and more stable self-concepts may facilitate adaptive responses to stressors commonly encountered in older adults, including health-related challenges or social transitions. This pattern therefore highlights that aging is not uniformly associated with vulnerability, but can also be characterized by psychological resources and adaptive regulation (87) that may be associated with fewer gambling-related difficulties.

### 4.2 Predicting the GD severity

The model obtained for predicting GD severity among the clinical subsample (N = 745) identified global psychological distress (as measured by the SCL-90R GSI) as the most influential predictive variable. This finding highlights the association between GD severity and overall psychopathological burden, and is consistent with previous research showing that higher levels of psychiatric symptoms (88, 89), including personality pathology (90), are associated with more severe addictive behaviors, greater functional impairment, and poorer clinical outcomes. In adults aged 50 to 85 years, this association may be particularly salient, as psychological distress often reflects the cumulative impact of chronic stressors, health-related concerns, and emotional challenges accrued across the lifespan. However, the cross-sectional nature of the present study does not allow determining whether psychological distress contributes to greater GD severity or is partly a consequence of more severe gambling problems.

Novelty seeking again emerged as an important predictive variable, indicating that impulsive–exploratory tendencies were associated with both the presence of GD and greater severity within the clinical subsample (23, 31). The persistence of novelty seeking as a severity-related variable in this age group suggests that reward sensitivity and impulsivity-related traits may remain relevant to the intensity of gambling involvement well into older adulthood, although causal or longitudinal relationships cannot be inferred. Gambling-related cognitive distortions also played a prominent role, supporting cognitive-behavioral models that emphasize the importance of erroneous beliefs about chance, illusion of control, and biased interpretations of wins and losses in gambling-related problems (17, 23). In older adults, such distortions may be reinforced by accumulated gambling experiences and selective recall of past wins, contributing to the consolidation of maladaptive beliefs that sustain more severe and persistent gambling patterns.

The “*higher-severity*” pattern associated with greater predicted severity was characterized by elevated global psychological distress, stronger gambling-related cognitive distortions, engagement in strategic forms of gambling, tobacco use, and male gender. This pattern is consistent with prior evidence suggesting that, in older adults, gambling disorder severity may be associated with the convergence of emotional vulnerability, maladaptive cognitive processes, and co-occurring characteristics, rather than from isolated symptoms alone (17). Older men, in particular, have been reported to show higher lifetime exposure to gambling activities and a greater likelihood of persistent or severe gambling problems compared with women, a pattern repeatedly observed in epidemiological studies and systematic reviews of older adult populations (5). Gender-related psychosocial factors may also be relevant in this context, as transitions associated with retirement, reduced work-related identity, shrinking social networks, and increased social isolation can intensify emotional stress while simultaneously limiting access to adaptive coping strategies in older adulthood. When combined with cultural norms that encourage risk-taking, competitiveness, and perceived skill mastery —particularly among men— these factors may be associated with continued engagement in strategic or skill-based gambling activities. Such forms of gambling have been related to cognitive distortions such as illusion of control and biased interpretations of outcomes, which may contribute to loss of control and contributing to more severe and persistent manifestations of gambling disorder in older adults. Other studies have linked stronger cognitive distortions, particularly beliefs in luck and illusion of control, to problematic gambling and gambling disorder (91).

In contrast, the patterns associated with lower predicted severity (“*psychosocially stable lower severity*” and “*adaptive lower-severity*”) shared features of more adaptive personality functioning, lower psychopathology, and greater psychosocial stability. Variables such as being married, later onset of gambling problems, higher self-directedness and cooperativeness, and lower levels of psychological distress were associated with lower predicted severity, pointing to the potential relevance of both internal resources and social context in relation to GD severity (92). In older adults, these characteristics may be particularly relevant. Stable social relationships, such as long-term partnerships, can provide emotional support, practical guidance, and social monitoring that may be associated with lower levels of gambling behavior. Similarly, well-developed self-directedness and cooperativeness may reflect accumulated life experience and mature coping skills, which may support the management of impulses, regulate emotions, and navigate age-related stressors more effectively. Later onset of gambling problems may also co-occur with stronger cognitive and psychosocial reserves built over the lifespan, which can buffer against the intensification of symptoms. Overall, these findings suggest that, even in older adults, psychological resources and stable social contexts may be relevant to differences in GD severity.

### 4.3 Comparison between MLP y classical regression models (logistic and ordinal)

Overall, the comparison between binary logistic and ordinal regression models and the MLP showed substantial agreement regarding the main predictors associated with GD identification and severity. Rather than identifying a completely different set of predictors, the neural network largely converged with the classical models on the most relevant variables while potentially capturing additional predictive information arising from complex multivariate relationships among predictors.

These findings support a complementary interpretation of the two approaches. Whereas logistic and ordinal regression provide transparent and readily interpretable mathematical frameworks for quantifying the independent contribution of individual predictors, the MLP is designed to accommodate more complex multivariate structures and may therefore capture information that is not fully represented by conventional regression coefficients. In addition, the neural network provides descriptive insights into predictor importance and latent activation patterns that may be useful for generating new hypotheses regarding the mechanisms underlying both the presence and severity of GD.

Taken together, the findings suggest that the value of the MLP does not lie in identifying entirely different predictors from those detected by conventional regression models, but rather in its ability to model potentially complex relationships among these predictors while producing a comparable overall pattern of results.

### 4.4 Clinical implications

The present findings may have clinical relevance for the assessment and treatment of GD in older adults. However, despite the strong discriminative performance of the classification model, its potential clinical utility remains preliminary and requires replication and external validation before it can inform clinical decision-making.

First, the strong discriminative performance of the classification model suggests that psychological and personality variables, particularly novelty seeking and emotion regulation difficulties, may warrant further investigation as potentially informative variables for early identification. The present findings indicate that these factors were useful in distinguishing older adults with GD from healthy controls in our sample; however, future research is needed to determine their incremental value in broader clinical populations and relative to established gambling screening approaches.

Second, the identification of distinct psychological patterns associated with the presence of GD supports the need for case formulation–driven interventions. For individuals characterized by the “*impulsive–exploratory dysregulation*” pattern, treatment could consider placing greater emphasis on impulse control, behavioral planning, and strengthening self-directedness. In contrast, those fitting the “*emotion-driven dysregulation*” pattern could potentially benefit from greater emphasis on emotional awareness, acceptance, and adaptive emotion regulation strategies. Approaches such as cognitive-behavioral therapy enriched with emotion regulation components, mindfulness-based strategies, or transdiagnostic treatments may be particularly well suited for this subgroup.

The severity model further underscores the importance of addressing global psychological distress and gambling-related cognitive distortions in treatment. The strong association between overall psychopathological burden and GD severity highlights the potential relevance of interventions that do not focus exclusively on gambling behavior but also actively assess and treat comorbid symptoms of depression, anxiety, and general distress. Integrated or sequential treatment models that address both GD and co-occurring emotional disorders may therefore be worth considering for older adults, who often present with complex clinical patterns.

The relevance of cognitive distortions highlights the continued centrality of cognitive restructuring techniques in this age group. Challenging erroneous beliefs about control, probability, and gambling outcomes may be particularly relevant among individuals engaged in strategic forms of gambling, where illusions of skill may be especially reinforced; although older adults who prefer strategic gambling are less commonly seen in clinical settings, it is important to consider their needs and ensure the availability of tailored intervention programs for this group. Conversely, the identification of lower-severity pattern characterized by higher self-directedness, cooperativeness, and psychosocial stability may identify potentially relevant targets for treatment and future relapse-prevention research, rather than established protective factors.

The finding that sociodemographic variables played a comparatively minor role in both models suggests that clinicians should avoid over-relying on demographic risk markers when assessing GD in older adults, reinforcing the importance of nuanced psychological assessment. A comprehensive evaluation focusing on personality style, emotion regulation, cognitive distortions, and overall psychological distress may provide a potentially informative basis for individualized treatment planning.

### 4.5 Strengths and limitations

#### 4.5.1 Final del formulario

This study has several strengths. A key contribution is the use of neural network models, which allow the examination of complex, non-linear relationships among psychological, personality, and clinical variables. This approach made it possible to identify not only individual predictors but also meaningful psychological patterns that may not have emerged from more traditional methods. Another strength is the comprehensive, multidimensional assessment, encompassing temperament and character traits, emotion regulation, general psychopathology, gambling-related cognitions, and sociodemographic variables. This broad perspective is particularly valuable in older adults, in whom GD often co-occurs with emotional distress and age-related psychosocial changes. Finally, focusing specifically on adults aged 50 to 85 addresses an underrepresented population in GD research, contributing novel knowledge about risk and protective factors in older adults.

However, several limitations should be acknowledged. First, the cross-sectional design precludes causal inferences and prospective prediction. Although several demographic, clinical, and psychological characteristics were strongly associated with GD presence and severity, it is not possible to determine whether these associations reflect antecedents, consequences, or bidirectional processes. Longitudinal studies are needed to clarify temporal relationships, examine how these psychological patterns evolve over time, and determine whether they have predictive value for the subsequent development or progression of GD. Moreover, although model performance was evaluated across training, testing, and holdout subsets, the present study does not provide independent external validation or establish the model’s calibration, incremental value over existing assessment approaches, or clinical utility. Therefore, the observed discriminative performance should not be interpreted as evidence that the models are ready for clinical implementation. Furthermore, although formal sample size calculations based on the framework proposed by Riley et al. (67) indicated that the overall sample size was adequate relative to the number of candidate predictors, the marked imbalance between GD cases and healthy controls resulted in a relatively small number of controls available for holdout validation. Consequently, specificity estimates are likely to be less precise and more variable than sensitivity estimates, and some degree of model optimism cannot be entirely ruled out despite the use of early stopping, holdout validation, and repeated random-split validation. Prospective studies in independent clinical populations are needed to establish the robustness, generalizability, and potential clinical usefulness of the models.

Second, while neural networks are powerful predictive tools, their complexity can limit interpretability. Although the analysis of hidden neurons provided clinically meaningful patterns, these representations remain indirect and should be interpreted cautiously. Replication using alternative modeling approaches, such as penalized regression or decision tree–based methods, would help to confirm the stability of the identified predictors and patterns.

A further limitation is that the UPPS-P and GRCS were not administered to the HC group. Although these measures could have provided dimensional information on impulsivity and gambling-related cognitions among controls, their absence precludes direct between-group comparisons for these constructs and should be considered when interpreting the corresponding findings.

Another limitation is the long recruitment period (2014–2025). Although the same assessment instruments and procedures were used throughout the study, changes in gambling products, online gambling availability, public awareness, and treatment pathways during this period may have introduced potential period or cohort effects. However, an analysis of GD severity across recruitment years showed no significant differences in DSM-5 GD severity, *F*(10, 734) = 1.317, *p* = .217, providing no evidence of a systematic change in the primary clinical outcome over the recruitment period. The extended recruitment period also allowed us to obtain a large clinical sample, which is particularly important given the relatively low prevalence of gambling disorder in the age range examined, even in specialized treatment settings.

A limitation concerns the marked gender imbalance between the GD and HC subsamples. Gender was deliberately not included as an input variable in the classification model because, given the substantial imbalance between groups, it could have provided a strong group-discriminating signal that would have been difficult to disentangle from GD-specific characteristics and could have increased the influence of sample-composition effects on classification. However, this decision does not eliminate the possibility that gender-related differences are indirectly represented through the psychological variables included in the model. Therefore, we cannot rule out that some of the model-derived patterns partly reflect gender-related differences rather than characteristics specifically associated with GD. Future studies using gender-balanced samples and independent clinical comparator groups are needed to address this issue.

Finally, the generalizability and clinical specificity of the findings may be limited by sample characteristics and recruitment sources. The clinical sample consisted of treatment-seeking patients with GD, whereas HC participants were recruited from individuals attending hospital-based Dentistry and Podiatry services rather than through population-based sampling. Although both groups were recruited from the same geographical catchment area, the present design does not allow us to determine whether the model specifically identifies GD-related characteristics or, in part, differences associated with treatment-seeking status or broader psychological distress. The model was not tested against older adults with other psychiatric disorders, substantial psychological distress without GD, or problematic gamblers who do not seek treatment. Future studies including these clinically relevant comparator groups will be necessary to establish the specificity and clinical utility of the model as a GD risk-discrimination tool.

### 4.6 Conclusion

The identification of distinct patterns associated with higher or lower likelihood of GD underscores the heterogeneity of gambling-related patterns in older adulthood. In adults aged 50 to 85 years, the psychological characteristics associated with GD in the present sample appear to reflect the interplay between enduring individual characteristics and age-related contextual and developmental factors, rather than from aging per se. Although some patterns were characterized by impulsive or affective dysregulation, others were characterized by adaptive personality functioning and emotional regulation. This framework is consistent with a lifespan-oriented view of GD in which older adulthood represents neither a uniformly high- nor low-risk period, but rather a developmental stage in which different patterns of vulnerability and adaptive functioning may be observed depending on psychological resources and contextual demands. Importantly, this multidimensional framework may provide a useful basis for understanding not only the presence or absence of GD in older adults, but also variations in symptom expression and severity.

The findings of this study suggest that GD in older adults is associated with a range of psychological characteristics, including psychological distress, personality traits, emotion regulation difficulties, impulsivity, and cognitive distortions. These findings support the potential value of comprehensive clinical approaches that extend beyond behavioral control of gambling to include interventions targeting emotion regulation, comorbid psychopathology, maladaptive gambling-related beliefs, and coping strategies for stress.

## Data Availability

The datasets analyzed during the study are not publicly available due to patient confidentiality. Requests to access these datasets should be directed to the corresponding author.
